# Copper toxicity and deficiency: the vicious cycle at the core of protein aggregation in ALS

**DOI:** 10.3389/fnmol.2024.1408159

**Published:** 2024-07-09

**Authors:** Jin-Hong Min, Heela Sarlus, Robert A. Harris

**Affiliations:** Department of Clinical Neuroscience, Karolinska Institutet, Center for Molecular Medicine, Karolinska University Hospital at Solna, Stockholm, Sweden

**Keywords:** ALS, copper, protein, aggregate, TDP-43, SOD1, C9ORF72, neurodegeneration

## Abstract

The pathophysiology of ALS involves many signs of a disruption in copper homeostasis, with both excess free levels and functional deficiency likely occurring simultaneously. This is crucial, as many important physiological functions are performed by cuproenzymes. While it is unsurprising that many ALS symptoms are related to signs of copper deficiency, resulting in vascular, antioxidant system and mitochondrial oxidative respiration deficiencies, there are also signs of copper toxicity such as ROS generation and enhanced protein aggregation. We discuss how copper also plays a key role in proteostasis and interacts either directly or indirectly with many of the key aggregate-prone proteins implicated in ALS, such as TDP-43, C9ORF72, SOD1 and FUS as well as the effect of their aggregation on copper homeostasis. We suggest that loss of cuproprotein function is at the core of ALS pathology, a condition that is driven by a combination of unbound copper and ROS that can either initiate and/or accelerate protein aggregation. This could trigger a positive feedback cycle whereby protein aggregates trigger the aggregation of other proteins in a chain reaction that eventually captures elements of the proteostatic mechanisms in place to counteract them. The end result is an abundance of aggregated non-functional cuproproteins and chaperones alongside depleted intracellular copper stores, resulting in a general lack of cuproenzyme function. We then discuss the possible aetiology of ALS and illustrate how strong risk factors including environmental toxins such as BMAA and heavy metals can functionally behave to promote protein aggregation and disturb copper metabolism that likely drives this vicious cycle in sporadic ALS. From this synthesis, we propose restoration of copper balance using copper delivery agents in combination with chaperones/chaperone mimetics, perhaps in conjunction with the neuroprotective amino acid serine, as a promising strategy in the treatment of this incurable disease.

## Introduction

Amyotrophic Lateral Sclerosis (ALS) is a heterogeneous neurodegenerative disease that primarily affects both upper and lower motor neurons, leading to their progressive degeneration and subsequent, often rapid loss of motor function and death (Brotman et al., 2022). Prognosis is very poor with a life expectancy ranging from 3 to 5 years from presentation of first symptoms, with the majority of deaths resulting from respiratory insufficiency (Niedermeyer et al., 2019; Masrori and Van Damme, 2020). ALS can be phenotypically classified into either limb onset (LO) cases with upper and lower motor neurons being affected, accounting for 70% of the cases, or Bulbar onset (25%) in which speech and swallowing issues first arise that are followed by later stage limb weakness (Zarei et al., 2015). ALS can also be genetically categorized as being either sporadic ALS (SALS) that represents 90–95% of all diagnoses and in which there is no known direct genetic contribution to the disease, or familial ALS (FALS) that makes up the remaining 5–10% of all cases where known genetic mutations such as in SOD1, TDP-43, FUS, and C9ORF72 predominate (Zarei et al., 2015). ALS is typically a late onset disease with a mean age of onset at 64 years old (Marin et al., 2016). However, cases of juvenile onset ALS (<25 years old) also emphasize the importance of genetic factors in contribution to the disease (Turner et al., 2012). Furthermore, sex is another factor, with males having a greater incidence of both SALS and FALS, the cause of this not yet being fully understood (McCombe and Henderson, 2010). The annual global incidence of ALS is 2.3/100,000 individuals, and in Europe and the USA its prevalence is between 5.2–6.2/100,000 individuals (Chiò et al., 2013; Xu et al., 2020). To date, there is no known cure for this disease and the only disease-modifying treatments currently approved are Riluzole and Edavorone, which only increase survival by 2–3 months or up to 6 months, respectively (Saitoh and Takahashi, 2020; Brooks et al., 2022).

The exact etiology of ALS remains unclear and has undergone many advancements since it’s conception as a purely motor neuron disease in 1869 (Masrori and Van Damme, 2020), although what remains central is the loss of function of the upper and lower motor neurons as described. In recent years much attention has been paid to the importance of genetic factors that contribute to ALS, that are all related protein aggregation in the spinal cord (Mejzini et al., 2019). Indeed, mouse models of ALS such as the well-used SOD1G93A and other models demonstrate that increased protein aggregation within the spinal cord is generally sufficient to induce symptoms that are the hallmark of the disease we call ALS, a fact that would place spinal cord protein aggregation as central to the disease (Todd and Petrucelli, 2022). This is not to exclude the importance of other aspects of ALS such as excitotoxicity, axonal transport issues, neuroinflammation and broader nutrient and metabolic deficiencies in ALS (De Vos and Hafezparast, 2017; Liu and Wang, 2017; Le Gall et al., 2020; Ludolph et al., 2023), though they are likely to be interlinked. Although we may touch on some of these issues in this review, we will focus on examining the pathways of protein aggregation and its downstream effects in the motor neurons for which exploration of the link between protein aggregation and pathological copper excess or deficiency is necessary (Posadas et al., 2023).

Copper imbalance can act twofold, firstly in excess as a cell stressor that can induce cuproptosis (Tsvetkov et al., 2022), and in deficiency in the loss of function of critical cuproenzymes that affect respiration and normal neuronal functioning (Hilton et al., 2024). This is most clearly demonstrated in the spinal cords of SALS patients as well as in SOD1G93A mice (Tokuda and Furukawa, 2016), showing that regardless of the cause in humans there is a loss of copper levels in the gray matter of the spinal cord and a decline in cuproenzyme function. There is also an increase in copper in the dorsolateral white matter columns that may also be subject to copper toxicity (Hilton et al., 2024), indicating that both toxicity and deficiency could happen simultaneously. It is therefore important to discuss the intricacies of copper metabolism, including how imbalance of this metal ion in the CNS leads to many of the hallmarks of ALS. In addition to this, we outline the functions of the major players in ALS such as superoxide dismutase 1 (SOD1), C9ORF72, TDP-43, and FUS, describing their general mechanisms and where known, their relationship with copper homeostasis and to a lesser degree their interactions with other metal ions. Finally, we discuss the known environmental risk factors and highlight how they participate in protein aggregation and their relevance to copper and protein aggregation. This is a key point as much of the cause of SALS appears to be environmental, as demonstrated by hotspots of increased ALS incidence or ALS risk-associated occupations in many countries (Caller et al., 2009; Henry et al., 2015; Goutman et al., 2022; Vasta et al., 2023). In this regard the most notable risk factors seem to be related with contamination of food or water sources with plant and algal toxins, and also heavy metals (Newell et al., 2022), all of which either enhance protein aggregation or interact with copper or cuproproteins in a detrimental manner (Dunlop et al., 2013; Jaishankar et al., 2014; Sheykhansari et al., 2018; Balali-Mood et al., 2021; Diaz-parga et al., 2021). In this review we attempt to synthesize the evidence to date into a coherent framework to outline the key mechanisms behind SALS and FALS, namely environmental causes that are compounded by genetic factors that lead to the disruption of proteostasis and cuproenzyme dysfunction that could fuel a vicious cycle of protein aggregation, further loss of cuproenzyme function in a terminal decline of motor neuron function known as ALS.

## ALS pathophysiology

The pathophysiology of ALS is characterized by the progressive degeneration and dysfunction of upper and lower motor neurons, although the exact mechanisms are unknown (Gordon, 2011). ALS can be due to heterogenous causes and subsequent heterogenous pathologies, which has made the exact determination of cause and development of effective treatment difficult (Hardiman et al., 2017; Tzeplaeff et al., 2023). In general, toxic protein aggregates form within the motor neurons that can cause RNA trafficking issues, mitochondrial damage, lysosomal dysfunction and synaptic signaling deficiencies (Hardiman et al., 2017). These aggregates are most commonly include of SOD1 aggregates, FUS, TDP-43 and C9ORF72, with both loss of normal protein function and formation of large aggregates that interfere with basic cellular processes (Hardiman et al., 2017). Vascular alterations occur before symptom onset, leading to increased hypoxia that stresses cellular metabolism (Nomura et al., 2019; Månberg et al., 2021). The combination of increased inflammation driven by microglia and release of toxic factors from astrocytes further stresses motor neurons, which includes elements of the complement system, pro-inflammatory cytokines such as tissue necrosis factor (TNF) as well as a reduced capacity of astrocytes to buffer extracellular glutamate that leads to neuronal glutamate excitotoxicity (Clarke and Patani, 2020; Yang et al., 2024). With regards to which toxic factors are specifically released by astrocytes, it is not fully known but may contain a cocktail of the aforementioned factors with other novel, yet to be identified mediators (Nagai et al., 2007). In the end, these combined factors contribute to the gradual degeneration and loss of executive motor functions in ALS patients (Haukedal and Freude, 2019; Vaz et al., 2021), a process which likely begins with the detachment of the motor neurons from the neuromuscular junctions in what is known as the dying back hypothesis (Verma et al., 2022).

Many of these functions that see decline in ALS are crucially dependent on copper including mitochondrial respiration, antioxidant defense, iron metabolism, neurotransmitter synthesis and vascular function (Uauy et al., 1987; Schuschke, 1997; Desai and Kaler, 2008). Furthermore, pathological unbound copper can from non-canonical copper interactions with ALS related proteins can influence other aspects of ALS such as protein aggregation, proteostasis and lysosomal function, which are the key areas of discussion in this review (Polishchuk and Polishchuk, 2016; John et al., 2021; Zuily et al., 2022). We will discuss the roles the major ALS related proteins such as SOD1, TDP-43, FUS, C9ORF72 as well disrupted systems such as the lysosomal and vascular systems and how they related to copper homeostasis. To begin, it is important to first of all discuss the role of main role of copper in the CNS and the problems when dysfunction arises.

## Copper regulation: engines of enzymes

Owing to the catalytic properties of copper it is incorporated into many key enzymes. For example, in the functioning of mitochondrial cytochrome oxidase C complex 4 (COX4) which facilitates electron transport in oxidative respiration, or in SOD1 which provides defense against free radicals, and in ceruloplasmin (CP) which regulates cellular iron content (Uauy et al., 1998). Insufficient loading of copper into these enzymes results in impaired function despite adequate tissue protein levels, and this is a feature evident in both mouse models of ALS and in patients (Hilton et al., 2018, 2024). In these enzymes copper is usually co-ordinated by either histidine or cysteine residues positioned in precise geometries to ensure specificity in their function (Inesi, 2017). A list of catalytic cuproenzymes and their functions in humans is summarized in Table 1.

**Table 1:** List of catalytic cuproenzymes in the human central nervous system.

Cuproenzyme	Abbreviation	Function
Cytochrome-c oxidase (Richter and Ludwig, 2003)	COX4	Electron transport, terminal oxidase
Superoxide dismutase 1 and 3 (Antonyuk et al., 2009; Trist et al., 2021)	SOD1&3	Superoxide dismutation
Lysyl oxidase (Rucker et al., 1998)	LOX	Collagen and elastin cross-linking
Ceruloplasmin (Linder, 2016)	CP	Ferroxidase
Hephaestin (Helman et al., 2023)	HEPH	Ferroxidase
Amine oxidases 2 and 3 (Finney et al., 2014)	AOC2/&3	Deamination of primary amines
Dopamine-b-hydroxylase (Rahman et al., 2009)	DBH	Dopamine → norepinephrine
Peptidylglycine monooxygenase (Bousquet-Moore et al., 2010)	PAM	α-Amidation of neuropeptides
Tyrosinase (Tief et al., 1998; Branza-Nichita et al., 1999)	TY	Melanin Synthesis

**Figure 1:**
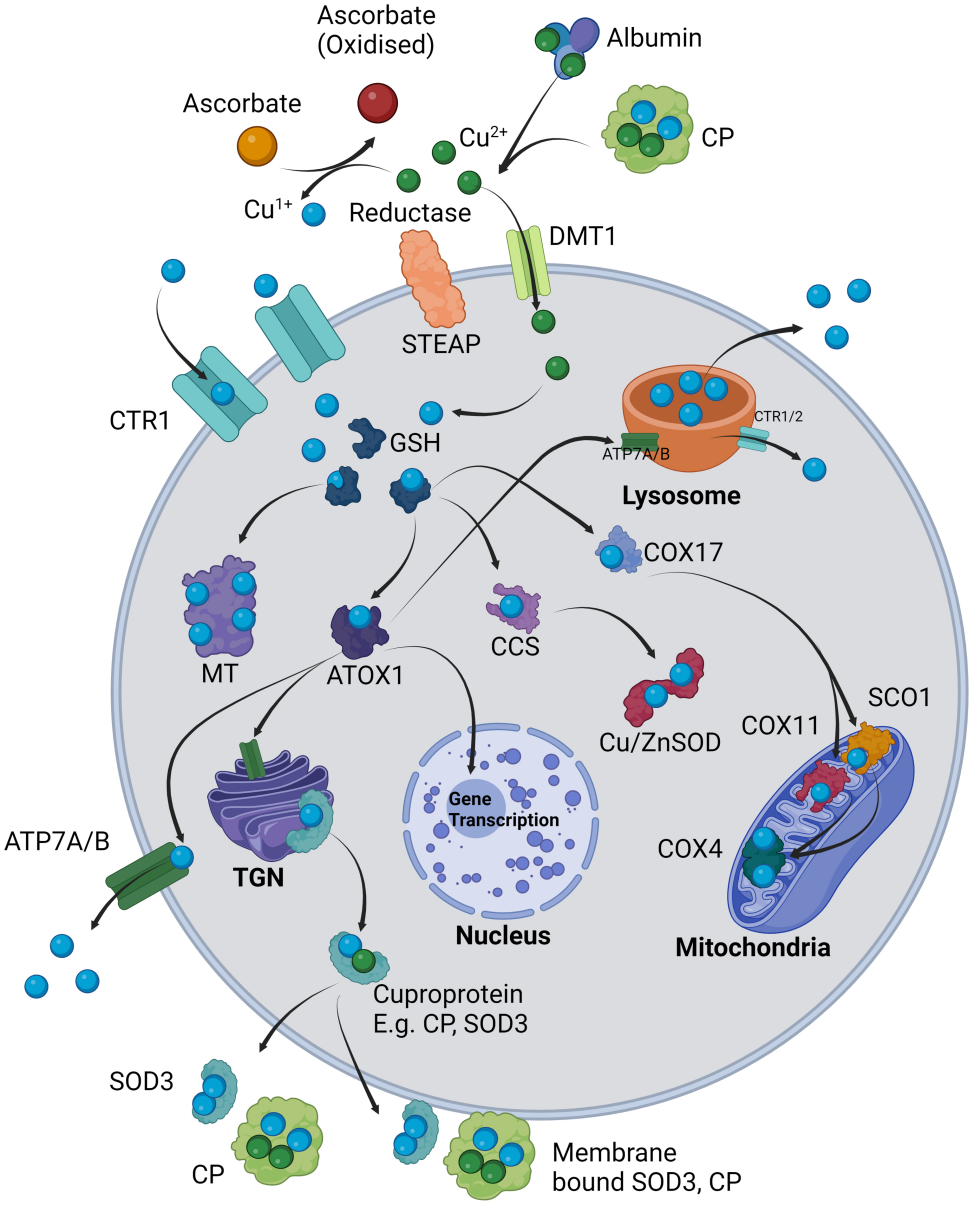
Cellular regulation of copper: Cu^2+^ is provided by carriers such as albumin and CP and in the presence of a surface reductase such as STEAP, the copper is liberated and reduced to Cu^1+^ in co-ordination with ascorbate and taken in through copper transporter 1 (SLC31A1/CTR1). Alternatively, Cu^2+^ is transported intracellularly via DMT1 where it is likely internally reduced to Cu^1+^. Cu^1+^ is immediately sequestered by glutathione (GSH) which delivers it to chaperones, e.g., ATOX1, CCS, COX17 or for long-term storage in MT. ATOX1 delivers Cu^1+^ to the TGN where it supplies copper for incorporation into exported cuproproteins such as CP or SOD3 which may also be bound on the cell surface. ATOX1 also delivers Cu^1+^ to ATP7A/B (depending on cell type) where it is either exported from the cell or in other cases loaded into the lysosomes. The lysosome can directly export copper extracellularly via exocytosis or release Cu^1+^ back into the cytosol via CTR1/2. ATOX1 may also enter the nucleus and initiate transcription of responsive genes. CCS primarily delivers Cu^1+^ to Cu/ZnSOD (SOD1). COX17 delivers Cu^1+^ to mitochondrial COX11 and SCO1, which in turn both provide Cu^1+^ to COX4.

The loading of these enzymes is directly related to cellular copper homeostasis. Copper is firstly usually bound to albumin or another transporter such as CP and is then delivered to the cell (Harris and Percival, 1989; Moriya et al., 2008). The primary mechanism for intracellular copper import is via solute-like carrier 31A1 (SLC31A1), otherwise known as copper transporter 1 (CTR1), which accepts reduced copper Cu^1+^ for transport. However, it must first be reduced by a metalloreductase such as prion protein (PrP), amyloid precursor protein (APP), or members of the six transmembrane epithelial antigen of prostate (STEAP) family of metalloreductases, usually in conjunction with ascorbate, from Cu^2+^ to Cu^1+^ for cellular import (Hellman and Gitlin, 2002; Ohgami et al., 2006; Prohaska, 2008; Scarl et al., 2017). Once inside the cell, copper is quickly bound to glutathione (GSH) with which it becomes redox-inactive and can be further processed for storage in metallothionines (MT), or incorporation into chaperones as antioxidant 1 (ATOX1), CP, SOD1, COX4 through the help of molecular chaperones that stabilize the apo-enzyme and load it with copper (Prohaska, 2008; Santoro et al., 2020). Alternatively, Cu^2+^ can enter via divalent metal transporter 1 (DMT1) where it is likely rapidly converted to Cu^1+^ in the reducing environment of the cytosol (Koch et al., 2003; López-Mirabal and Winther, 2008). Cu^1+^ can also be transported into the lysosome via lysosomal ATP7A/B, where it acts as a storage site for copper that can be exocytosed in bulk to reduce intracellular copper loads, or otherwise exported back into the cytosol via CTR1/2 (Polishchuk and Polishchuk, 2016). ATOX1 can deliver Cu^1+^ to the trans Golgi network (TGN) where it incorporates copper into plasma membrane or export-bound proteins such as extracellular and cell surface bound SOD3, CP (both extracellular and cell surface bound), andalso delivers copper to the nucleus for gene transcription of, e.g., vascular endothelial growth factor (VEGF) and LOX (Hatori and Lutsenko, 2016; Liu Z. et al., 2022). The copper chaperone for superoxide (CCS) delivers Cu^1+^ to SOD1 for free radical scavenging (Schmidt et al., 2000). Cytochrome c oxidase copper chaperone 17 (COX17) delivers Cu^1+^ to cytochrome c oxidase 11 (COX11) and cytochrome c oxidase assembly protein (SCO1), that both finally deliver Cu^1+^ to COX to enhance oxidative respiration (Ruiz et al., 2021). Copper export from the cell can also be governed by ATP7A and ATP7B, that in conjunction with CTR1 help maintain the copper flux within the cell (Prohaska, 2008; Scheiber et al., 2014). This maintenance is summarized in Figure 1.

In the CNS, copper metabolism is mainly governed by astrocytes whose end feet surround the blood brain barrier (BBB). These cells have a great ability to buffer metal ions and are coupled to neurons in their copper metabolism (Scheiber et al., 2014).

## Copper and cell death: how does copper kill?

As excess copper is extremely dangerous, the amount of unbound copper in a cell is estimated to be at less than 0.1%, with one free copper ion per cell under normal conditions (Rae et al., 1999; Manto, 2014). This strict regulation is due to free copper being able to interact with proteins in a non-canonical manner, affecting their structure and generating ROS via the Fenton reaction (Manto, 2014; John et al., 2021). Copper deficiency has long been known to cause mitochondrial dysfunction, demyelination, iron overload and neurodegeneration, and so adequate provision of copper to cuproenzymes is important (Cobine et al., 1868; Benetti et al., 2010; Gulec and Collins, 2014; Prodan et al., 2023). When a cell fails at copper management, either due to total overload or overload due to vulnerability from diminished chaperoning and storage ability, the cell will become oxidatively stressed and protein aggregation (in a unique form of programmed cell death known as cuproptosis) may occur (Charbonnier et al., 2022; Tsvetkov et al., 2022; Zuily et al., 2022). It is therefore likely that failure of the chaperone system itself can simultaneously cause functional deficiency and toxic overload. In ALS there is an imbalance of copper, with a mix of both copper deficiency and overload which can be explained by the mis-partitioning of copper stores, with a loss of cuproenzyme function and increased amounts of copper-bound aggregates, which will be discussed later (Gil-Bea et al., 2017; Barros et al., 2018; Chen Q. Y. et al., 2022). While copper is a well-known inducer of cell death, until recently the exact underlying mechanism in mammalian cells was not fully elucidated. Upon exposure to toxic levels of copper, the copper binds to lipoylated TCA cycle proteins such as dihydrolipoamide S-acetyltransferase (DLAT), promoting the formation of disulfide bonds and toxic aggregation of this protein and other related TCA cycle proteins inside the mitochondria, this being concomitant with the loss of Fe-S clusters. The net result is the triggering of a mitochondria-dependent programmed cell death. An important note is that chelation of the copper ions via the antioxidant GSH effectively prevents cuproptosis, whereas non-copper chelating antioxidants such as N-acetylcysteine (NAC) and Ebselen have no effect, this indicating that the copper ions themselves and not ROS are critical for cuproptosis (Tang et al., 2022; Tsvetkov et al., 2022).

Copper can also cause long-term stress to the cell in concentrations that are not immediately lethal, one such effect being protein aggregation. The exact mechanism for how copper induces aggregation is complex and requires understanding of the effect of pH, charge of the copper ion and oxygenation. An interesting study investigating the mechanisms of protein aggregation using *E. coli* elucidated that copper can induce protein aggregation both under aerobic and anaerobic conditions, though not equally (Zuily et al., 2022). Under anaerobic conditions a far greater amount of aggregation occurs, which is attributed to increased intracellular import of copper. This imported copper can exist as either Cu^1+^ or Cu^2+^ which exert their aggregation effects in different ways. Firstly, proteins that form aggregates bound to Cu^1+^ are richer in histidine and cysteine residues than those formed with Cu^2+^, this likely being due to Cu^1+^ being a soft acid and reacting more with the thiol groups present on these amino acids to alter the protein into non-native conformation states. Secondly, Cu^2+^ works by generation of ROS that oxidize cysteine residues to form non-native bonds on affected proteins (Pearson, 1963; Zuily et al., 2022). An example of this is the binding of Cu^2+^ to ubiquitin to its aggregation-prone regions, destabilizing its structure and leading to aggregation and functional failure of this core component of the ubiquitin proteasome system (UPS; Arnesano et al., 2009). Cu^2+^ can also promote protein aggregation by oxidizing and forming intramolecular cysteine bonds between two separate peptides through inner sphere electron transfer in a catalytic process (Prudent and Girault, 2009). This catalysis might in part explain why Cu^2+^ promotes Parkinson’s disease-associated protein α-synuclein aggregation without altering fibril structure, and is likely a central mechanism by which protein aggregation is accelerated by free Cu^2+^ (Rasia et al., 2005).

Conversely, copper deficiency can result in loss of cuproenzyme function, most notably COX4 of the mitochondrial electron transport chain. Metabolic reprogramming via copper-modulated COX4 assembly governs mitochondrial turnover as well as cell differentiation, with copper promoting mitochondrial biogenesis and oxidative respiration whereas decreasing copper concentration will downregulate COX4 expression and promote glycolysis and proliferation (Ruiz et al., 2021). The deficiency of COX activity evident in ALS patient spinal cords may potentially be caused by functional copper deficiency (Fujita et al., 1996; Borthwick et al., 1999). This is possible due to either gross copper deficiency or chaperone deficiency, as not all cuproenzymes are equally affected in ALS (Hilton et al., 2018, 2024).

Overall, the general effects of systemic copper deficiency can be best exemplified in Menkes disease patients, who harbor a genetic disease resulting in mutation of the ATP7A transporter which leads to severe systemic copper deficiency (Tümer and Møller, 2010). Usually this results in eventual death from COX4 and SOD1 dysfunction and ROS overload (Ruiz et al., 2021). The effective loss of function of these two enzymes in Menkes disease is reflected by a similar pathology in ALS that would suggest shared pathological mechanisms that stem from functional copper deficiency. Furthermore, copper deficiency has also been shown to induce mitochondrial swelling, a feature also seen in SOD1 mutant motor neurons, indicating another possible link between copper deficiency and ALS (Vande Velde et al., 2011; Smith et al., 2019; Ruiz et al., 2021).

## Copper and the heat shock response

When cells encounter a variety of different stressors such as heat, osmotic, hypoxic, and oxidative stress, the heat shock protein (HSP) response is activated to generally enhance cell survival under such conditions (Hu et al., 2022; Jeyachandran et al., 2023), as well as the unfolded protein response (UPR) upon detection of the accumulation of misfolded or unfolded proteins (Hetz et al., 2020). Similarly, when cells are challenged with toxic levels of copper they also rapidly begin to upregulate a range of UPR and HSP related genes (Saporito-Magriñá et al., 2018). This is followed by the ubiquitin proteasome (UPS) and autophagy systems that work to combat the dangers of copper-induced protein aggregation. One of the major functions of the HSP system is in regulation of protein quality control by assisting in the proper folding of proteins as well as disaggregation of existing protein aggregates. HSP70 is quickly and highly upregulated during copper toxicity, which co-ordinates with other HSPs for disaggregation (Parsell et al., 1994; Duennwald et al., 2012; Mokry et al., 2015; Saporito-Magriñá et al., 2018). This indicates that protein quality control is of primary concern for cells that encounter a copper-related stressor.

In ALS, levels of elements of the HSP system (e.g., HSP70, HSP27 and HSP90) are significantly elevated in SALS patients and HSPs are also significantly elevated in the brains of C9ORF72-ALS/FTD patients (Miyazaki et al., 2016; Mordes et al., 2018). The importance of HSP27 and HSP70 is striking as they are more highly expressed in spinal motor neurons than in other neurons, suggesting an increased reliance of motor neurons on HSP systems (Mattson, 2002). Interestingly, HSPs themselves can be disabled by being sequestered in aggregates. For instance, SOD1 and HSPs occur together in aggregates in both SOD1 mutant mice spinal cords and in post-mortem ALS spinal cords (Mattson, 2002). This observation has led to the hypothesis that high levels of unfolded WT or unfolded mutant SOD1 can act by directly sequestering HSPs and depleting the cell of this valuable defense mechanism (Mattson, 2002; Kalmar et al., 2014). In order to understand more about how each of the major aggregate-prone proteins interact with copper and contribute to the resultant pathology, we will discuss the major protein dysfunctions and their related pathways involved in ALS.

## SOD1: β-sheets, metals, and aggregation

SOD1 is best known for its key role as an intracellular defense mechanism against ROS such as superoxide anions (O_2_^−^), whereby SOD1 catalyzes the dismutation of O_2_^−.^ Into H_2_O_2_ via the following reaction: 2O_2_^−.^ + 2H^+^ → O_2_ + H_2_O_2._ The majority of O_2_^−^ is localized within the cytoplasm, but is also found in the nucleus, peroxisomes and mitochondrial intermembrane space (Crapo et al., 1992; Nandi et al., 2019). Each SOD1 enzyme comprises of 2 β-barrel subunits that are held together by a disulfide bridge between cysteine residues 57–146 to form a dimer, with each monomer coordinating a copper ion between histidine residues that possess the redox cycling ability necessary for catalytic dismutation (Berdyński et al., 2022). Interestingly, SOD1 can also be translocated into the nucleus upon ROS generation sources such as H_2_O_2_ and paraquat (PQ), whereupon it acts as a transcription factor binding DNA and promotes antioxidant gene expression (Tsang et al., 2014).

Up to 20% of all FALS cases and therefore 1–2% of all ALS cases are directly linked to SOD1 mutations and the vast majority of these patients primarily experience motor symptoms (Gertz et al., 2012; Marangi and Traynor, 2015). The familial SOD1 mutations are also extremely varied, with 185 mutations currently having been identified, and molecular modeling has been used to predict the effects of many of these variants. The most severe variants such as D109Y, L126, N86s and G72S affect the metal ion binding or catalytic sites, while G37R, G41S, G93C affect the β-sheet organization, and K3E, D90A, D109Y and G37R modify the overall electrostatic charge. These varied mutations can thus contribute to structural disorder, loss of catalytic function and/or aggregation (Berdyński et al., 2022).

SOD1 is also innately liable to misfold and accumulate as aggregates for several reasons. Firstly, SOD1 contains multiple sequences in its amino acid structure that result in β-sheets which are prone to amyloid fibril formation and thus aggregation, a feature that is conserved in both SALS and FALS SOD1 (Ivanova et al., 2014). This is especially true for monomeric apo-SOD1, as in this state the central β-sheets are prone to unfolding and thus expose their binding sites (Jahan and Nayeem, 2020). Secondly, the stability of SOD1 is also determined by sufficient metalation of apo-SOD1 into holo-SOD1 with zinc and copper, which greatly enhances SOD1 structural stability and reduces SOD1 aggregate formation. Thirdly, the formation of oxidized disulfide bonds within the SOD1 structure between cysteine residues further strengthens the protein structure, whereas reduced disulfide bonds, as seen in apo-SOD1, facilitate misfolding and aggregation (Lelie et al., 2011; Sheng et al., 2012; Boyd et al., 2020).

This combined process of metalation, cysteine bond formation and progression from aggregation-prone monomer to more stable dimer is naturally achieved by the cognate chaperone for SOD1, CSS. CSS facilitates transport and loading of copper into the structure of SOD1 in the final stages of maturation into holo-SOD1, and also promotes the formation of disulfide bonds in the SOD1 structure, thereby stabilizing the enzyme through two mechanisms (Casareno et al., 1998; Luchinat et al., 2017). It is also noteworthy that in the CNS, SOD1 exists in a 12-30x molar excess compared to CCS (Rothstein et al., 1999), that presents the possibility of CCS insufficiency in SOD1-overexpressing models. This concept has led to the hypothesis of CCS availability in proportion to SOD1 as being a rate-limiting condition in effective SOD1 maturation and aggregation prevention, and hence it is a target of interest for therapeutic intervention (Williams et al., 2016). In addition to this, one study had reported that in ALS mice and human patient tissue, CCS was sequestered in all aggregates identified, which indicates a pressure on chaperoning and proper metalation capacity (Watanabe et al., 2001). This is further demonstrated by the evidence of mislocalisation of CCS along with dysfunctional SOD1 within ALS patient spinal cords (Trist et al., 2022b). It is important to note that overexpression of CCS in the mouse SOD1G93A model significantly exacerbated disease progression in these mice (Son et al., 2007), which has been strongly suggested to be due to an interference with mitochondrial function due to CCS overexpression induced copper deficiency (Son et al., 2008; Williams et al., 2016). We therefore emphasize that CCS would be beneficial only combined with copper supplementation to reduce the risk of further copper chelation.

The significance of improved metalation, specifically with copper, has been confirmed by several studies *in vivo*. Firstly, a study in SOD1^G37R^ mice demonstrated that overexpression of human CTR1 (hCTR1) is protective by increasing copper transport into neurons and restoring function to the accumulated pool of enzymatically inactive cuproenzymes (e.g., CP and SOD1; Hilton et al., 2018). Furthermore, delivery of copper to the CNS of SOD1^G93A^ mice using the bis(thiosemicarbazonaes) PET scanning agent Copper diacetyl-bis(N4-methylthiosemicarbazone; Cu-ATSM) led to increased survival and improved cognitive performance (Vieira et al., 2017; Lum et al., 2021). When combining increased CNS copper delivery and promoting disulfide bond formation, survival can be further enhanced, as is the case of SOD1^G93A^ mice that co-express human copper chaperone for superoxide (hCCS) (hCCS x SOD1^G93A^) in conjunction with CuATSM administration from birth. These mice had significantly increased spinal cord holo-SOD1 and survived for an average of 540 days, whereas normal SOD1^G93A^ mice survived an average of 130 days, representing an almost 4-fold increase in lifespan. In addition, the disease progression could be stopped and started by addition or withdrawal of Cu-ATSM, respectively, indicating its potential use not only in the prevention of the disease but also in its treatment (Williams et al., 2016; Molnar-Kasza et al., 2021). Conversely, simple exposure to copper without chaperones or delivery agents *in vitro* strongly enhances the aggregation of SOD1, so the correct chaperoning of copper is essential (Li et al., 2013).

In this regard, the copper homeostasis within the motor neurons themselves is of utmost importance and has been found to be disrupted in ALS patient spinal cords as well as in the SOD1G93A mouse model (Williams et al., 2016; Hilton et al., 2024). The effect we believe could be twofold, either through toxicity caused by the excess of redox active copper that is free to form harmful reactive oxygen species (ROS) that can cause damage through lipid peroxidation and DNA damage (Sauzéat et al., 2018; Sheykhansari et al., 2018), or by functional deficiency as previously mentioned, although the two may not be mutually exclusive.

One study using Chinese hamster ovary (CHO) cells modified to express four different SOD1 mutants indicated increases in metal free protein aggregates and a copper deficiency that demonstrates this aggregation process alone is sufficient to induce copper deficiency in a variety of mutants (Bourassa et al., 2014). It therefore seems most likely that it is a functional deficiency initiated by protein aggregates, or the aggregation process in motor neurons, that is central to ALS. To further support this, markers for cuproptosis could be measured in ALS spinal cords to see if copper toxicity is concomitant with existing known markers of functional deficiency (Tang et al., 2022). Further evidence from ALS patients indicates that SOD1 aggregates can accumulate both within neurons and astrocytes (Kato et al., 2000). It is unclear whether these astrocytic aggregates have a similar effect on the copper homeostasis of astrocytes, but given the effects of copper depletion in the neurons and the effects of the SOD1 aggregates in CHO cells, one can speculate that a similar copper depletion could be occurring within astrocytes. This is important as astrocytes are well known to be critical to the nutritional support of neurons and act as the primary copper (and other metal ion) buffer in the CNS (Dringen et al., 2013). Such a lowered buffering capacity and dysfunction would likely exacerbate copper handling in neurons and other cells of the CNS and may also be another mechanism by which CuATSM exerts its positive effects.

## TDP-43: cell stress, metals, and aggregation

TDP-43 is an RNA/DNA binding protein that is mostly expressed in the nuclei of all cell types during homeostasis, regulating transcription, translation and mRNA stability (Suk and Rousseaux, 2020). The most common targets of TDP-43 binding are RNAs that code for neuronal survival, development and regulation of synaptic proteins, with the net result of maintaining these mRNA levels (Polymenidou et al., 2011; Tollervey et al., 2011). However, TDP-43 can form aggregates that have been observed in 97% of ALS and 45% of frontotemporal lobar dementia (FTLD) cases, representing a large fraction of ALS aggregates that can be considered a hallmark of this disease (Suk and Rousseaux, 2020).

The formation of TDP-43 aggregates is linked to (but not entirely dependent on Fernandes et al., 2020) its ability to form cytoplasmic stress granules upon oxidative stress, heavy metal exposure, hypoxia, heat shock, viral infection or osmotic stress (Khalfallah et al., 2018). These stress granules temporarily halt protein translation in order to protect mRNA that will be released upon removal of the stressor, thus allowing normal translation to resume (Protter and Parker, 2016). In this regard TDP-43 is recruited to form a part of stress granules where it is essential in maintaining stress granule structural integrity (Khalfallah et al., 2018). These granules exist as a membrane-less organelle, in part due to the ability of TDP-43 to undergo liquid–liquid phase separation (LLPS). This process involves the assembly of TDP-43 into liquid droplets that likely assist its ability to maintain the phase separation of the stress granule to form isolated liquid compartment within the cell which houses the fragile mRNA (Babinchak et al., 2019).

However, under conditions of prolonged stress TDP-43 in the stress granules will accumulate in a dissociation-resistant gel form. This is in part explained by a structurally important observation that TDP-43 contains an intrinsically disordered prion-like domain that at neutral pH can self-assemble into β-sheet-rich oligomers (Lim et al., 2016; Suk and Rousseaux, 2020). If chronically stressed, these TDP-43 aggregates can persist even after removal of the stressor and dissolution of the stress granules and contribute to the aggregate load in the cells (Parker et al., 2012a; Ratti et al., 2020). The effects of genetic mutations of TDP-43 identified in FALS and SALS can vary, from increasing the fragmentation propensity and neurotoxicity (such as in Q331K and M337V mutants) and also excess stability, for instance through increased cysteine disulfide bond-forming regions in G348C mutants that promote aggregation (Kabashi et al., 2008; Sreedharan et al., 2008; Ling et al., 2010).

A wide range of viruses including human endogenous retrovirsues (HERVs), severe acute respiratory syndrome coronavirus 2 (SARS-CoV-2) and herpes simplex virus-2 (HSV) and enterovirsues (EV) have all been shown to interact with TDP-43, which is unsurprising due to its role in RNA regulation (Rahic et al., 2023). The first characterization of TDP-43 was from its inhibitory effects on HIV-1 replication, thus indicating this response as a potential mechanism of combating viral infection (Ou et al., 1995; Rahic et al., 2023). Although a later study challenged this notion and showed no inhibitory effect of TDP-43 on HIV replication, a recent study reported that TDP-43 overexpression negatively impacts HIV viral fusion and inhibits infection (Nehls et al., 2014; Cabrera-Rodríguez et al., 2022). In addition, Coxsackievirus B3 (CVB3) increased the translocation of nuclear TDP-43 to the cytoplasm, leading to formation of TDP-43 aggregates. However, upon TDP-43 knockdown, viral titres were increased (Fung et al., 2015; Xue et al., 2018b), suggesting the protective role of TDP-43 against viral infections. This relationship between viral infections and TDP-43 may be connected as copper is linked to LLPS induction in general, a topic that will be discussed later.

Metal ion balance is significantly altered in ALS patient CSF and blood, with significant increases in manganese, copper, aluminum, cadmium, zinc, lead, vanadium and uranium (Roos et al., 2013). Lead and mercury salts increase TDP-43 aggregation *in vitro* (Ash et al., 2019) and zinc directly induces TDP-43 aggregation due to its binding to RNA recognition sites and decreasing TDP-43 thermostability, an effect not evident with either copper or iron (Caragounis et al., 2010; Garnier et al., 2017). Cadmium works differently by competitively displacing zinc from its binding site in SOD1, thereby leading to protein folding defects and inactivating the enzyme while simultaneously generating ROS (Huang et al., 2006; Oggiano et al., 2021). Together, this stresses the importance of environmental metal ion toxicity as a contributing factor to ALS.

Metal ions can also contribute to ROS production by directly participating in redox reactions. Numerous studies indicate occupational exposure and residence proximity to areas treated with agrochemicals, in particular herbicides and pesticides, as being a significant risk factor for developing ALS (Bonvicini et al., 2010; Kamel et al., 2012; Malek et al., 2012; Andrew et al., 2021). A well-studied example is the herbicide paraquat (PQ; mentioned earlier for its effects on SOD1) that is better known for the relationship between exposure and risk of developing Parkinson’s Disease (PD) and its use in inducing mouse models of PD (Che et al., 2018; Tangamornsuksan et al., 2019). PQ exerts its toxicity via ROS generation by redox cycling, a mechanism which has been demonstrated *in vitro* to induce TDP-43 aggregation (Parker et al., 2012a; Gao et al., 2020). Interestingly, an earlier study on rice leaves indicated this toxicity is mediated by interaction with copper and iron ions that decrease SOD1 activity, with decreased toxicity when chelating away these ions (Chang and Kao, 1997). However, a later *in vitro* study showed that in contrast, copper in conjunction with ionophores such as Cu-ATSM or glyoxal-bis(N4-methylthiosemicarbazone) (Cu-GTSM), can prevent paraquat-induced stress granule formation, TDP-43 aggregate formation and cytotoxicity, thus indicating the protective role of adequate intracellular copper loading in preventing TDP-43 mislocalisation and aggregation (Parker et al., 2012b).

Although the exact mechanisms of the interaction between TDP-43 and copper are not well understood, TDP-43^A315T^ transgenic mice that express a familial mutation of TDP-43 demonstrate an increase in spinal cord metal ion levels, notably zinc, manganese and copper (Dang et al., 2014). Interestingly, these three divalent metal ions have also been shown to be involved in prion diseases (Leach et al., 2006). The exact reason for this elevation of these ions has yet to be determined, but this study does indicate the ability of aberrant TDP-43 to induce metal ion dysfunction that can pressure metal ion buffering systems with pathological consequences. Conversely, studies of the direct interaction between copper and TDP-43 are lacking, but it appears that elevated copper levels are insufficient to induce TDP-43 aggregation in cells *in vitro* whereas zinc is able to induce this aggregation (Caragounis et al., 2010). The effects of copper on TDP-43 are thus likely much less direct than in the case of copper on SOD1, but conversely TDP-43 defects appear to significantly increase divalent metals ion levels including copper (Dang et al., 2014). Furthermore, TDP-43 can co-aggregate with canonical copper binding enzymes such as SOD1 that could inactivate them and thus contribute to functional cuproenzyme deficiency (Trist et al., 2022a). As such, TDP-43 induction through a variety of mechanisms could induce aberrant metal ion and copper distribution and thus contribute to the disturbed copper levels we see in ALS patients spinal cords (Hilton et al., 2024).

## FUS: cell stress, DNA repair, and manganese superoxide dismutase

Similarly to TDP-43, FUS is another DNA/RNA binding protein that is involved in the cell stress response. It is responsible for DNA damage repair, DNA stability, RNA transcription, transportation and alternative splicing and is mainly localized inside the nucleus (Yang et al., 2014; Ishigaki and Sobue, 2018). Upon DNA damage FUS forms a liquid–liquid phase separated zone around the damage site to recruit DNA repair enzymes and maintain them at high concentration, and facilitates DNA repair in a compartment separate from the rest of the cell (Patel et al., 2015). However, FUS can also be localized to the cytoplasm to participate in stress granule formation. Here it can co-localize with TDP-43, this being governed by intrinsically disordered prion-like domains (PLDs; Sama et al., 2013; Aulas and Vande, 2015).

FUS mutations are the second most common in FALS (3.2% of all FALS; Blair et al., 2010). These mutations primarily affect the ability of FUS to remain in a soluble liquid–liquid phase and enhance transition into liquid solid phase fibrous aggregates with mutation loci on their PLDs impairing protein translation (Dutertre et al., 2014; Patel et al., 2015). FUS also contains zinc finger domains that participate in its recognition of RNA (Wang et al., 2015), and the NIH protein database predicts that human FUS exhibits metal ion binding ability (FUS FUS RNA binding protein, 2023). Although it is currently unknown whether zinc can directly influence FUS aggregation, owing to the similarities between FUS and TDP-43 in form and function, it is possible that metal ions interact with FUS and promote its aggregation in a similar way as they do with TDP-43 (Garnier et al., 2017), although this remains to be experimentally verified. However, it has recently been shown that metal ions including zinc and copper can facilitate the LLPS of FUS that has been modified with an N-terminal hexahistidine tag, a modification that was used to demonstrate the influence of metal ion-histidine interactions in LLPS activity of proteins (Li et al., 2022). Further studies could be performed to *in vitro* to directly elucidate the effect of metal ions such copper and zinc in the propensity of wild-type and ALS risk associated variants of FUS to LLPS and ultimately FUS aggregation.

Aside from the direct effects on copper, FUS has also been shown to be a transcription regulator governing the expression of manganese super oxide dismutase (MnSOD; Dhar et al., 2014). This is important as MnSOD is the main form of SOD present in the mitochondria where it exerts superoxide dismutase activity with arguably greater overall importance than Cu-Zn-containing SOD1 (Holley et al., 2011). This is succinctly demonstrated by the inability of SOD1 over-expression to prevent the neonatal lethality of MnSOD deficiency, whereas SOD1 knockout in mice has not been shown to be directly lethal (Copin et al., 2000; Saccon et al., 2013). In FALS patients FUS mutations have been identified that result in lower systemic levels of MnSOD that are suggested to lead to slow and cumulative oxidative damage in mitochondria (Dhar et al., 2014). This is relevant as a lack of MnSOD activity can exacerbate symptoms and speed up mortality in SOD1G93A mice that could also be due to increased pressure on overall ROS dismutase activity and mitochondrial health (Andreassen et al., 2000). Increased levels of MnSOD seen in presymptomatic SOD1G93A rats has been postulated to be a compensatory mechanism for the loss of functional SOD1 (Stamenković et al., 2017), an increase that is also evident in the ALS patient spinal cords (Liu et al., 1998). Therefore, whatever the cause of disruption in FUS signaling such as aggregation (Shelkovnikova et al., 2014), there will be downstream effects on the ability to contribute to the overall SOD activity of the cell. The disruption of both of these elements of SOD activity evident in ALS may be a key element in understanding the role of FUS in ALS, via the shared overlap between copper and manganese dependent systems governed by SOD1 and FUS-regulated MnSOD.

## C9ORF72: haploinsufficiency and aggregate toxicity

Given that C9ORF72 mutations are the most frequent mutation in ALS, FALS and FTD, understanding the function of the gene and the effects of its mutation have been extensively investigated (Gijselinck et al., 2012; van Blitterswijk et al., 2013; Maharjan et al., 2017; Gossye et al., 2020). The mutation itself usually consists of expanded hexanucleotide repeats (HRE) consisting of multiple G_4_C_2_ repeats in the non-coding region of C9ORF72 (Reddy et al., 2013). These HREs can be detrimental by (i) haploinsufficiency, where the RNA itself fails to be translated properly, resulting in lowered C9ORF72 protein levels and (ii) by translation of dipeptide repeat proteins (DPR) which are neurotoxic and contribute to degeneration of neurons in the CNS (Shi et al., 2018). Furthermore, carriers of C9ORF72 HREs are associated with shorter survival. In order to understand how haploinsufficiency and translation of dipeptide repeat proteins (DPR) contribute to ALS, we must first investigate the homeostatic functions of C9ORF72.

C9ORF72 is a multifunctional protein that is an important regulator of vesicle trafficking, autophagy, RNA transport and localization, nuclear DNA damage repair and cytoskeletal organization, and is highly expressed in myeloid cells and neurons (Smeyers et al., 2021; He et al., 2023). Like TDP-43 and FUS, C9ORF72 plays a prominent role in SG regulation. C9ORF72 co-localizes with aggregates of messenger RNA ribonuclear proteins called P-bodies and also co-localizes with SGs, and reduction in C9ORF72 leads to inhibition of the SG assembly (Parker and Sheth, 2007; Maharjan et al., 2017). C9ORF72 is mainly located in the nucleus where it is involved in DNA damage repair (Maharjan et al., 2017; He et al., 2023). The DNA and RNA processing capability is similar mechanistically to that of TDP-43 and FUS, as C9ORF72 also undergoes LLPS owing to its PLDs that are rich in polar uncharged amino acids (e.g., asparagine, glutamine and glycine; King et al., 2012; Boeynaems et al., 2017).

C9ORF72 interacts with Rab-GTPases and regulates the trafficking of vesicles between different cellular compartments including endosomal transport, autophagy and lysosomal biogenesis (Smeyers et al., 2021). In myeloid cells the deletion of C9ORF72 leads to exocytosis of lysosomal enzymes and impaired lysosomal function, which in turn leads to an inflammatory response and tissue damage (Smeyers et al., 2021). Cultured stem cell-derived neurons from C9ALS/FTD patients display dysregulation in Rab signaling and reduced autophagy (Webster et al., 2018; Zhang et al., 2018).

In addition to these functions, C9ORF72 is highly expressed in myeloid cells such as monocytes, microglia and dendritic cells (O’Rourke et al., 2016; Smeyers et al., 2021), and has been shown to regulate the activity of STING (stimulator of interferon genes), a major regulator of immune responses. STING plays a key role in the detection of viral and bacterial pathogens and triggers the production of type I interferons and other cytokines (Ahn and Barber, 2019; Kabelitz et al., 2022). Deletion of the C9ORF72 gene in myeloid cells in mice led to hyperactivation through increased production of type 1 interferons and conditions reminiscent of autoimmune diseases such as lymphoid hypertrophy and splenomegaly (McCauley et al., 2020). This is suggested to result from decreased autophagic degradation of STING. The increase in type 1 interferons is also seen in C9ORF72 ALS and FTD patients, supporting the critical role of C9ORF72 in repressing excessive inflammation in myeloid cells in FTD and ALS (McCauley et al., 2020).

Aside from haploinsufficency, the toxic translated DPRs also contribute not only in terms of aggregate formation, but also by reducing proteasome function. This is achieved by poly-GA aggregates recruiting large numbers of the 26S proteasome which is structurally altered and impaired in function (Guo et al., 2018). It is important to note that copper positively enhances 26S proteasome function degrading CCS, although the exact mechanism is not fully understood (Bertinato and L’Abbé, 2003). Overall, the direct relationship between copper and C9ORF72 has not been established, but given the ability of copper to induce protein aggregation in a variety of neurodegenerative proteins it would be beneficial to directly assay this *in vitro.* However, the most important impact that C9ORF72 aggregation has on copper homeostasis may be related to its propensity to aggregate. In particular the C9ORF72 poly-GR aggregates have been shown to induce TDP-43 aggregates (Cook et al., 2020). The interactivity of these protein aggregates is important with regards to copper homeostasis, a topic that shall be discussed in the next section.

## Metal ions, LLPS, and co-aggregation of ALS proteins

The similarity between each of these ALS associated proteins is that they all undergo LLPS, including SOD1 (Gu et al., 2023). This is because intrinsically disordered domain-containing proteins are naturally susceptible to aggregation (Ayyadevara et al., 2022), and also to metal ion-included LLPS (Sołtys et al., 2023). In particular, copper and zinc can induce LLPS as seen in AD with both Aβ and Tau undergoing LLPS in response to metal ion-induced stress (Fu et al., 2024). In PD α-Synuclein also undergoes LLPS via copper exposure (Ray et al., 2020). Furthermore, even IDPs on mosquito receptors undergo LLPS induced by copper ions (Więch et al., 2021). Many viruses also utilize LLPS within the infected cell to enhance their replication and therefore have been implicated in neurodegenerative diseases, such processes again being regulated by copper and zinc (Monette and Mouland, 2020). In this regard copper and zinc both display strong intracellular antiviral abilities, which may explain why certain families of DNA viruses contain decoy SOD1 homologs that can bind and render copper ions inactive (Monette and Mouland, 2020; Rani et al., 2021). The connection between viral infection, LLPS, metal ion homeostasis and neurodegeneration thus serves to address the association of viruses with ALS (Celeste and Miller, 2018; Xue et al., 2018a; Bellmann et al., 2019). While it appears that excess or displaced metal ions (especially copper and zinc) can act as initiators of LLPS, and thus aggregation of IDPs, it has yet to be demonstrated in all the aforementioned ALS-related proteins.

Although ALS-related proteins and their propensity to aggregate due to metal ions has been discussed, it is important to note the complex interplay between these proteins in forming aggregates and conversely the effect that this has on copper homeostasis. Interestingly, TDP-43 and FUS can both induce prion-like seeding and misfolding of SOD1, potentially through the release of naked aggregates or within disease-associated exosomes from stressed neurons (Pokrishevsky et al., 2016). This cross-protein aggregation therefore raises the issue that such as the seeding of aggregation prone proteins such as SOD1 in ALS. This would therefore demonstrate an interplay between the effect of excess copper on promoting the aggregation of certain proteins, but also explain why TDP-43 and FUS aggregates exacerbate functional copper deficiency, namely by sequestering and disabling SOD1 into aggregates and thus further reducing the total copper binding capacity of the cell. This resultant lowered copper binding capacity most probably explains the severe copper depletion seen in the aforementioned SOD1 mutant cell lines (Bourassa et al., 2014), and depletion of copper in the gray matter in human SALS spinal cords (Hilton et al., 2024).

**Figure 2:**
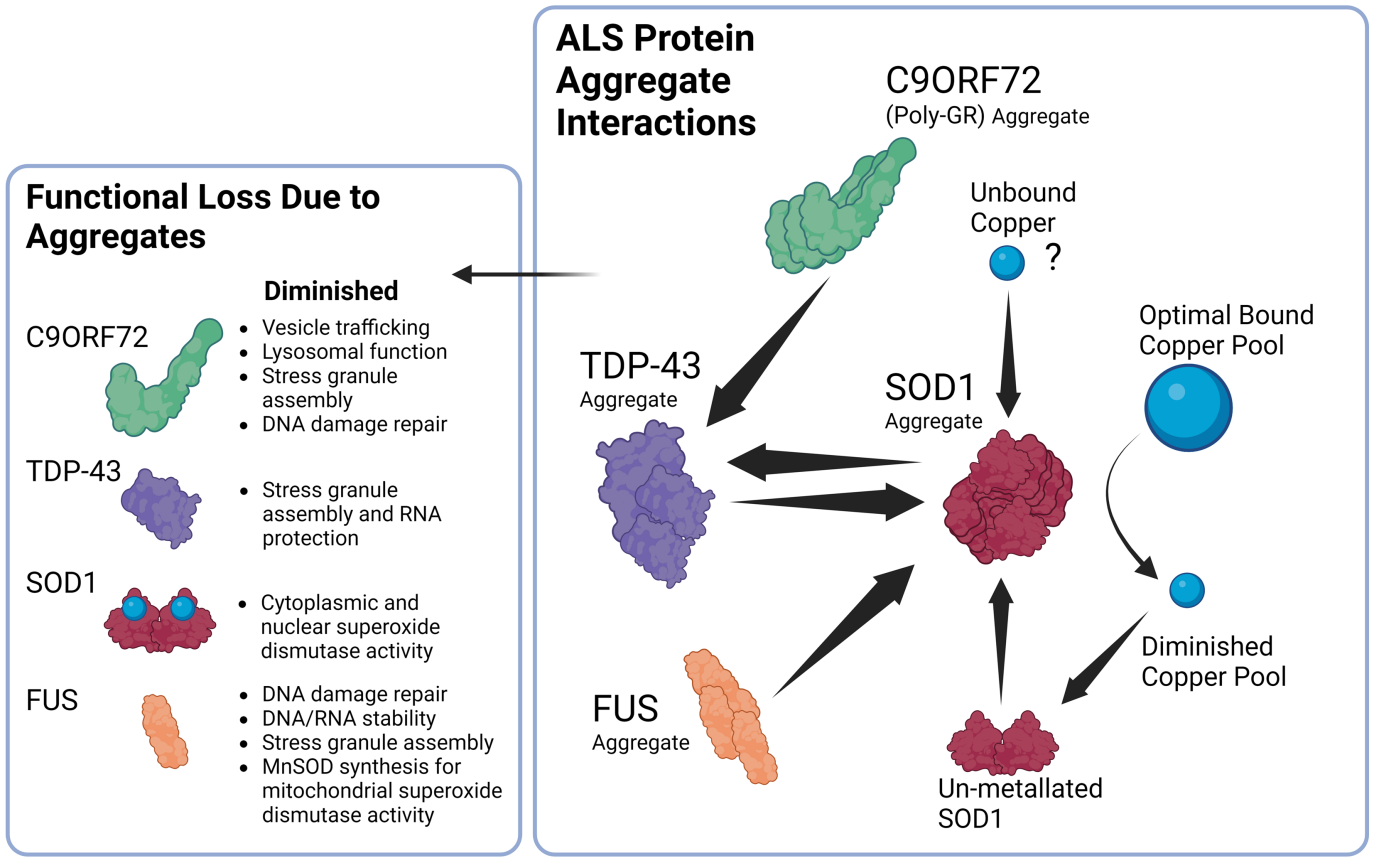
Proposed Interaction between ALS related proteins aggregates and the downstream consequences of dysfunction. Beginning with the right panel, SOD1 aggregates can be induced by TDP-43 and FUS aggregates, likewise TDP-43 aggregates can in return be induced by SOD1 aggregates establishing a potential feedback loop. In addition, C9ORF72 Poly GR aggregates induce TDP-43 aggregation that can again contribute to SOD1 aggregation. SOD1 aggregates lead to the diminished copper buffering capacity of the cell and ultimately reduced bound copper levels in the cell, diminished copper levels trigger SOD1 synthesis that results in un-metallated aggregation-prone SOD1 that contribute to further SOD1 aggregation. Unbound copper may possibly serve as a trigger to begin the cycle of aggregation due ability to trigger SOD1 aggregation.

Interestingly, copper depletion could also perpetuate a vicious cycle of aggregation, as copper depletion has been demonstrated to lead to upregulation of SOD1 protein synthesis as a response to reduced SOD1 activity that results in elevated levels of unmetallized non-functional SOD1 (Arciello et al., 2011). Ironically, the elevated levels of non-stable apo-SOD1 would be vulnerable to further seeded aggregation that perpetuates the cycle of copper deficiency and aggregation (Sheng et al., 2012). Taken together, it has therefore been argued that SOD1 misfolding is a key feature of not only of SOD1 FALS but also of SALS (Pokrishevsky et al., 2016; Paré et al., 2018). Interestingly, this aggregation can also occur in reverse, as exogenous SOD1G93A aggregates can cause aggregation and propagation of TDP-43 between neurons, thus further adding to the vicious cycle (Zeineddine et al., 2017). Hypothetically, a sufficient aggregation catalyst such as unbound copper (Capanni et al., 2004), or else an environmental toxin such as β-N-methylamino-L-alanine (BMAA; Ra et al., 2021), viral infection (Xue et al., 2018a), heavy metal toxicity (Ash et al., 2019) or a combination could initially seed the misfolding of one of these aggregation-prone species in the supersaturated cytosol to trigger a chain reaction and resultant vicious cycle that will gradually end in copper depletion and loss of cuproenzyme function that is characteristic of ALS. A simplified summary of the ALS protein aggregate interactions is illustrated in Figure 2.

## Lysosomes: copper and acid

Lysosomes are organelles responsible for the degradation of cellular waste products or foreign materials encountered through endocytosis, phagocytosis or autophagy. Lysosomes are often dysfunctional in many neurodegenerative diseases such as ALS, AD, PD, and MS (Bonam et al., 2019; Root et al., 2021). Lysosomes function by creating an enclosed membrane-bound space separated from the cytosol with a low pH of around 4.5–5. The pH is maintained by vacuolar-type H^+^ translocating ATPases (v-ATPase) which pump protons into the lysosomal lumen in an ATP-dependent manner (Mindell, 2012). The low pH is essential for the functioning of 60 different hydrolytic enzymes that include nucleases, lipases, proteases and others (Bonam et al., 2019).

Lysosomes are also a major storage site of copper, an important function that is tightly regulated. Copper staining with fluorescent copper sensor 1 (CS1) reveals strong co-localisation with lysosomes, indicating that it is a primary site of copper accumulation in the cell (Price et al., 2012). This is further supported by lysosomes expressing CTR1 and ATP7A and ATP7B which are all differentially regulated to modulate lysosomal copper concentrations (Polishchuk and Polishchuk, 2016). Lysosomes also act as a sensor of copper levels and will initiate lysosomal exocytosis of copper from cells if too high levels are detected (Peña et al., 2015; Polishchuk and Polishchuk, 2016).

Another major role of lysosomal copper is as an antimicrobial agent through enhancing ROS generation. When copper ions are present in lysosomes at low pH in the presence of H_2_O_2_, the copper ions are predominantly in the Cu^1+^ state and are thus more efficient in catalyzing the formation of hydroxide radicals (Xing et al., 2018). Although detrimental to the cell if left uncontrolled in the cytosol, this reaction is functional against pathogens in the lysosome. For example, LPS and IFN-γ stimulation enhance CTR1 expression on macrophages for intracellular copper import and ATP7A upregulation for copper trafficking to phagosomes to facilitate bactericidal activity through catalysis of H_2_O_2_ and HO• formation (White et al., 2009). However, in the absence of copper the bactericidal activity of macrophages was reduced by almost 80% (White et al., 2009). Another study reported similar findings with *Salmonella typhimurium* whereby copper accumulation was essential for a robust response against this intracellular pathogen (Achard et al., 2012).

The role of copper in lysosomal enzyme function is a poorly studied field. However, an important enzyme, acid sphingomyelinase (ASM), is known to utilize copper as a co-factor in the catalytic degradation of sphingomyelin. Mutations in this protein cause Niemann–Pick disease, characterized by lipid waste accumulation in lysosomes (Qiu et al., 2003). More research is needed to understand whether other enzymes require copper as cofactors and also to determine the charge state of copper in these dysfunctional enzymes.

The result of mutations in C9ORF72 or any ALS-related gene that disrupts proper lysosomal functioning will affect the pH and functionality of copper within the system, thus further impairing waste product degradation. Conversely, dysfunction in the copper chaperoning or buffering systems will affect functioning of lysosomal proteins. For example, Cu^2+^ has specifically been shown to inhibit V-ATPase activity which leads to decreased H^+^ trafficking, thus illustrating the importance of tight regulation of cytosolic copper (Miner et al., 2019). It is noteworthy that free Cu^2+^ can interact with anionic phospholipids and be reduced to Cu^1+^, whereby it binds to form a copper-lipid complex that stabilizes and stiffens the structure, affecting not only V-ATPase but also other membrane-bound organelle function (Garcia et al., 2005; Miner et al., 2019). Furthermore, given the importance of copper in the functioning of the lysosome, the ALS-related copper deficiency would have a detrimental effect on proper lysosomal functioning.

## Hypoxia: copper, HIF-1, and vasculature

This aspect of hypoxia has been established in the SOD1^G93A^ mouse model in which mutant mice have baseline impaired vascular endothelial growth factor (VEGF) in their spinal cords that preceded symptom onset. These mice also have an impaired ability to upregulate VEGF in response to hypoxia, demonstrating the effect of this aggregate-prone mutant in disrupting vascular signaling, an effect that is also seen in the G37R and G85R mutants (Murakami et al., 2003; Zhong et al., 2008). More recent studies in SOD1^G93A^ mice further indicate a severely reduced spinal cord blood flow and glucose transport as a result of this vascular impairment (Miyazaki et al., 2012).

In hypoxic situations the body responds by upregulation of vascular endothelial growth factor (VEGF) in order to increase vascular supply to the affected areas, which is governed by the hypoxia-inducible family of genes (HIF; Liu et al., 1995; Pugh and Ratcliffe, 2003). HIF-1 is comprised of two subunits, HIF-1α and HIF-1β, that together are necessary for HIF-1 functioning. HIF-1α serves as the rate-limiting subunit that is continuously degraded via hydroxylation by prolyl-4-hydroxylase domain enzymes (PHD) and subsequent recognition and degradation by the UPS system under normoxic conditions (Wang et al., 1995; Huang et al., 1998). However, during hypoxia HIF-1α is no longer degraded and forms a dimer with HIF-1β in the nucleus to form the HIF-1 transcriptional complex on the hypoxia-responsive element (HRE) of target genes such as VEGF (Semenza, 2001). This process is also regulated by the protein factor inhibiting HIF-1(FIH-1) that serve as negative regulators by preventing interaction between the two subunits and co-factors, thus inhibiting transcription (Lando et al., 2002). The interplay between degradation and successful dimerization is directly controlled by the interaction of copper and its chaperones in the HIF system. Copper is necessary for this function and may be required for HIF-1 binding to the HRE, as depletion of copper completely blocks this binding and prevents VEGF expression (Feng et al., 2009). Furthermore, this activity of copper is dependent upon CCS, as CCS directly interacts with the HIF-1α and silencing of the CCS gene prevents HIF-1 activation (Jiang et al., 2007; Feng et al., 2009). Copper also functions by inhibiting the degradation of the HIF-1α subunit by inhibition of PHD enzymes, and FIH-1 also stabilizes the subunit even under normoxic conditions (Martin et al., 2005; Feng et al., 2009). The handling of copper intracellularly is thus an integral part of the downstream HIF-1 signaling pathways, including VEGF. In order to maintain this increased demand for copper during hypoxia, the primary intracellular copper transporter copper transporter 1 (CTR1) is upregulated by HIF-1 activity in an autoregulatory fashion (Zimnicka et al., 2014).

In ALS a high degree of co-aggregation may explain part of the proteotoxic stress. Members of the HSP family as well as CCS are included in SOD1, TDP-43 and FUS aggregates (Ciryam et al., 2017; Trist et al., 2022a). This indicates that sequestration of these chaperones and anti-aggregation proteins are one mechanism by which expanding aggregates reduce the ability of cells to adequately meet cellular signaling demands. Furthermore, hypoxic stress induces the aggregation of WT-SOD1 (Woo et al., 2021), which would suggest a vicious cycle of SOD1 aggregation and sequestration of CCS, HSPs and other chaperones, leading to decreased proteostasis, decreased HIF-1-VEGF signaling and even more hypoxia and more SOD1 aggregation. We can deduce that SOD1 aggregates themselves are the initiators of this hypoxic cycle as overexpression of this mutant protein in SOD1^G93A^ mice results in vascular insufficiency. Attempts at restoring vascular deficits to the affected areas using the vasodilator ONO-1301-MS led to neuronal survival but had no effect on the overall life expectancy of ALS mice (Tada et al., 2019). The copper deficiency evident in SALS would only further inhibit HIF signaling due to the necessity of copper as a cofactor, and therefore this lack of copper would prevent proper vascularisation and an impairment of the hypoxic response. This demonstrates yet another arm of the positive feedback cycle of protein aggregation and functional copper deficiency.

## Upper vs. lower motor neuron differences in handling copper

Although motor neurons are affected in ALS a distinction must be made between the phenotype of upper and lower motor neurons, as this greatly affects the interpretation of pathologies. Upper motor neurons (UMN) extend from the motor cortex in the brain descending via the lateral corticospinal tract in the dorsolateral white matter (DLWM) of the spinal cord, where they then synapse with lower motor neurons (LMN) that are located in the anterior horn gray matter (Zayia and Tadi, 2022). An interesting observation in ALS patient spinal cords indicates the LMN dense gray matter is strongly deficient in copper and that the UMN dorsolateral white matter exhibits signs of copper overload (Hilton et al., 2024).

Neuronal presynaptic vesicles are often loaded with copper and are released into the synaptic cleft upon stimulation, where they act acutely to block postsynaptic channels and thereby downregulate signal transmission. This is reversed if chronic exposure to copper occurs, as increases in copper in the postsynaptic neuron leads to increased surface translocation but not expression of the GluA1 subunit of AMPA, and ultimately increases AMPA receptor density (Opazo et al., 2014). In ALS post-mortem samples the LMN AMPA receptors are also highly upregulated in all brain regions, with SALS patients expressing significantly higher levels of GluA1 mRNA. However, one exception to this is in C9ORF72 patients, whose spinal cords only have increased AMPA receptor expression (Selvaraj et al., 2018; Gregory et al., 2020). The mechanisms for this upregulation in ALS are not well studied, but the implications are increased excitotoxicity for affected neurons that could be exacerbated by copper-induced AMPA receptor clustering on the cell surface (Opazo et al., 2014; Gregory et al., 2020).

Apart from receiving AMPA signals, lower motor neurons are acetyl cholinergic in their signaling as they interface directly with muscle fibers at the neuromuscular junction (Zayia and Tadi, 2022). Interestingly, two case reports of severe copper deficiency resulted in LMN degeneration that had strong similarities to ALS (Weihl and Lopate, 2006; Benkirane et al., 2022). This is significant, as experiments in male rats show that sufficient dietary intake of copper resulted in increased acetylcholine (Ach) levels that were concomitant with an increased vasodilatory response and vascular smooth muscle relaxation, the opposite of which is true with copper deficiency (Schuschke et al., 1999). Furthermore, copper deficiency has long been known to cause blood vessel macromolecular leakage and is crucial in vascular functioning (Schuschke, 1997). These observations indicate the insufficient vascular supply in SOD1^G93A^ mice and highlights the importance of adequate copper supply.

ALS patient samples display excessive levels of copper in the DLWM (corresponding to the UMNs), high levels of insoluble copper, lower cuproenzyme activity in the whole spinal cord, and reduced levels of copper in the LMN gray matter (Hilton et al., 2024). Intuitively, UMN copper may be mislocalilzed and driven toward participating in aggregation, leading to functional copper deficiency. This could then lead to an interrupted supply of copper post-synaptically, resulting in LMNs that are copper deficient. As a result, both motor neurons would be affected and gradual loss of cuproenzyme activity would result in their death. It would be of benefit to experimentally clarify where gray matter LMNs receive the bulk of their copper supply from. This aspect of mispartitioning of copper may have been overlooked in the past, as this study found bulk spinal cord copper levels were not different from controls (Hilton et al., 2024). However, it is important to note that the spinal cord and brain have the slowest turnover of copper of any organ, indicating that whatever mispartitioning may happen, it will be slow to resolve (Levenson and Janghorbani, 1994).

## Conclusion and future perspectives

From the evidence presented herein we demonstrate the importance of copper in ALS, and how different commonly observed ALS-associated proteins such as SOD1, TDP-43, FUS and C9ORF72 are implicated in the disease pathology. Importantly, we have outlined the effects they each have on the ability to maintain proper copper homeostasis. The fact that copper is so precisely regulated renders the system vulnerable to even minor disruptions that over a long period of time that can lead to overall cellular failure and death.

**Figure 3:**
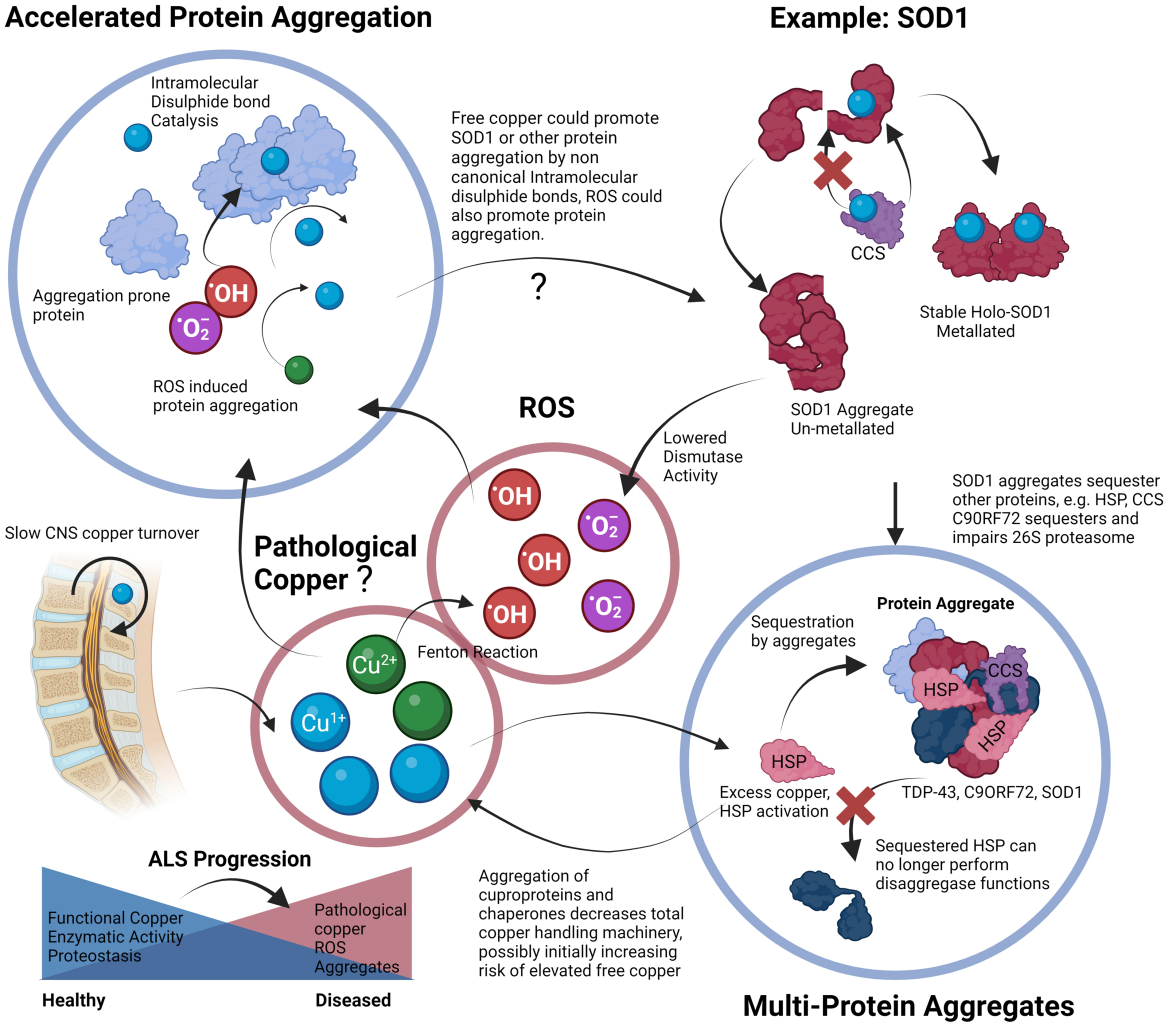
Proposed vicious cycle of copper, cuproprotein dysfunction and protein aggregation in ALS. Free copper could participate in the Fenton reaction to create hydroxyl radicals, these radicals and free copper could accelerate aggregation of aggregation prone proteins, especially cysteine rich proteins. Proper metalation of Apo-SOD1 to Holo-SOD1 by CCS promotes stable functioning SOD1 (or other cuproprotein with cognate chaperone). Lack of CCS activity, or proper incorporation and the effects of free copper enhance SOD1 aggregation. This lowered dismutase activity enhances superoxide anion levels that can further contribute to protein aggregation. Aggregates of SOD1 can further incorporate other proteins such as its own chaperone CCS that further enhances SOD1 aggregate formation, SOD1 can also accumulate HSPs that prevent dis-aggregation, thereby hampering proteostasis and leading to increases in aggregates. Although excess copper triggers HSP system activation, the effect of other aggregates to incorporate them neutralizes their protective effects. Aggregates are also multi-proteins, C9ORF72 can sequester 26 s proteasome subunits and inactivate them, thereby preventing protein degradation, TDP-43 also co-aggregates and may recruit other aggregation-prone proteins and functional proteins. Overall, excessive protein aggregation could result in elevated free copper levels due to lowered functional copper binding capacity, this free copper is then able to participate in the cycle again, and all systems dependent on copper will be affected. Slow CNS copper turnover could lead to retarded resolution of any imbalances, including of free copper. The proposed vicious cycle of copper disturbance and its relation to protein aggregation could be an important factor to ALS progression.

We have also discussed some of the genetic factors that include mutations in genes such as FUS, TDP-43, SOD1 and C9ORF72. Mutations in these genes already make aggregation-prone proteins susceptible to toxic changes that induce either a gain or a loss-of-function. Each of these proteins plays a specific role in the cellular proteostasis and stress response, and disruption of each synergistically affects the ability of a cell to handle copper-induced protein aggregation stress. From transgenic animal models of ALS in which the only difference is the mutant gene, it is plausible that it is the attenuation of the proteostatic systems themselves that are upstream in the pathological chain that leads to a vulnerability to copper-induced cell aggregation (Morrice et al., 2018). The cells could be left in a state in which they are unable to utilize copper properly due to a deficient functional state. We also propose that the “phenoconversion” (de Carvalho, 2022) of a pre-symptomatic patient to a symptomatic one occurs when the sum of the factors that enhance aggregation, such as ALS-related genetic mutations and environmental toxins, exceed the body’s capability to regulate proteostasis, which may be exacerbated by factors such as old age or nutrient deficiency (Hipp et al., 2019; Goncharova et al., 2021). We propose that the end-result is an insufficiently mitigated vicious cycle in which protein aggregation leads to copper deficiency and functional loss of copper-based enzymes, and proteostatic mechanisms that lead to more aggregation, a process that may possibly involve contribution from redox active pathological copper (Figure 3).

In order to further determine the role of copper in aggregation, more studies can be performed to identify the inclusion of either copper or copper chaperones and binding proteins within the ALS aggregates, as copper may act as a seeding core as for Aβ (Sasanian et al., 2020). Furthermore, although technically challenging it may be possible to understand the human copper-aggregatome by treating samples with toxic levels of copper, removing the insoluble precipitate fraction, re-solubilizing it by removal of copper and running proteomic analysis to identify which proteins collected are prone to aggregation, provided that the proteins are not permanently aggregated.

Interestingly, copper may be one reason for the observed sex difference in ALS susceptibility, one study reporting that healthy male subjects had serum copper levels of around 1 μg/mL and for females it was 1.2 μg/mL, a roughly 20% higher level in women (Buxaderas and Farré-Rovira, 1986). This elevation in systemic copper supply may be part of the reason why being female reduces ALS risk yet gives increased risk for MS, due to the previously discussed inflammatory association with copper (Strecker et al., 2013; Magyari, 2016; Liu Y. et al., 2022). Another interesting observation is that iron levels increase in the motor cortex and spinal cord in ALS patients (Bhattarai et al., 2022), furthermore there is evidence of reduced ferroxidase activity in ALS patient spinal cords despite elevated CP and hephaestin indicating these proteins are dysfunctional (Hilton et al., 2024). This is reminiscent of PD iron accumulation in the substantia nigra again due to dysfunction of copper-based enzymes such as CP (Ayton et al., 2013). Furthermore, CP is also lacking copper and is dysfunctional in AD patients, which is probably linked to the iron accumulation evident in AD patients (Brewer et al., 2010; Gleason and Bush, 2021). In each of these neurodegenerative diseases CP is dysfunctional and subsequent iron accumulation is evident (Vassiliev et al., 2005).

**Figure 4:**
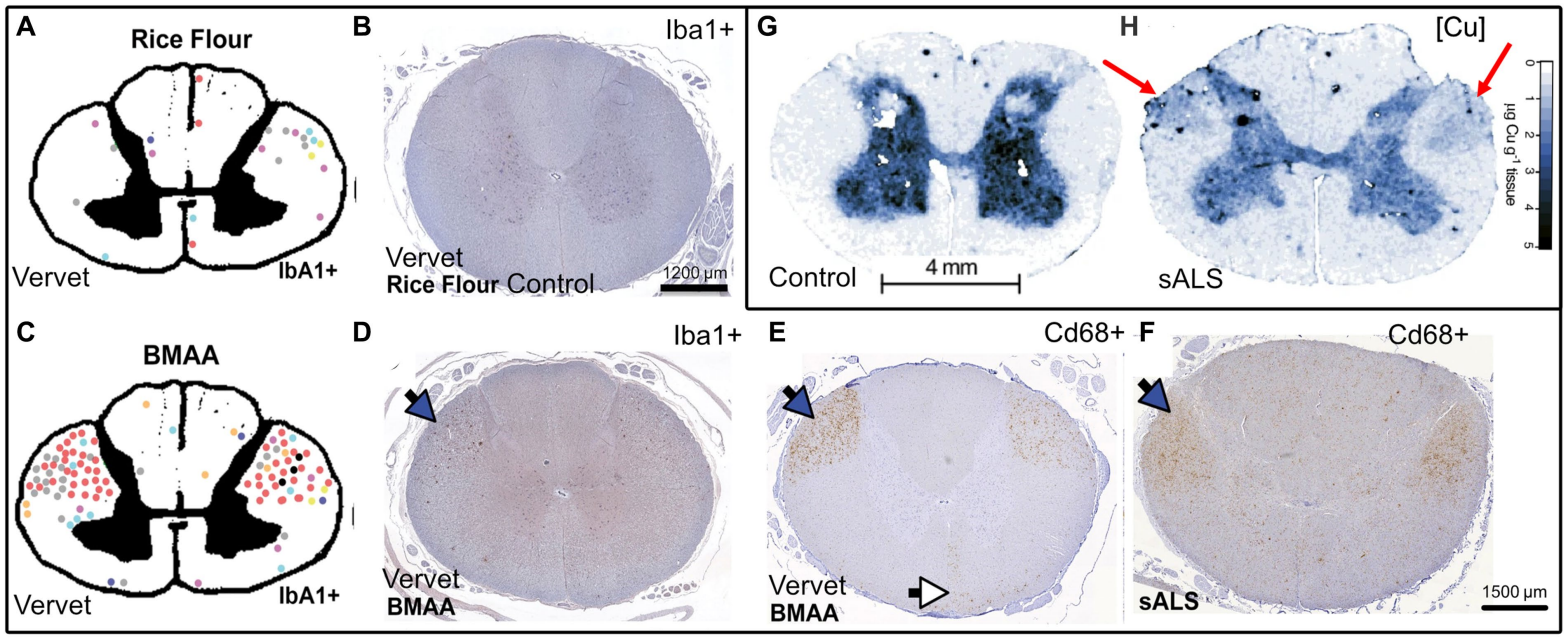
Qualitative comparison between BMAA-induced microglial activation in the spinal cord of vervet monkeys, SALS patients, and SALS spinal cord copper distributions synthesized from Davis et al. (2020) **(A–F)** and Hilton et al. (2024) **(G,H)**. **(A,C)** Pictorial representation the vervet monkey spinal cord with each colored dot representing a large area of Iba1 activation or nodule corresponding to an individual vervet monkey **(A)** = rice flour control 140 days, **(C)** = BMAA 210 mg/kg/day, 140 days, **(B)** Rice flour fed control vervet monkey spinal cord stained with Iba1 (microglia), **(D)** BMAA fed vervet monkey spinal cord stained with Iba1, **(E)** BMAA fed vervet monkey spinal cord stained with Cd68 (myeloid activation marker), **(F)** SALS patient spinal cord stained with Cd68, **(G)** Control patient spinal cord Cu levels (determined by LA-ICP MS), **(H)** SALS patient spinal cord Cu levels (determined by LA-ICP MS). Blue arrows represent the lateral corticospinal tracts and the white arrows represent the anterior corticospinal tracts, red arrows represent areas of the dorsolateral white matter that have become enriched with copper, scale bar in panel **(B)** applies additionally to **(D,E)**, Permission for re-use of figures has been granted by the respective authors.

The question remains as to what are the causes of ALS beyond the genetic risk factors? Since the major the ALS mutations are generally not considered fully penetrant (Chen et al., 2013; Murphy et al., 2017; Chen Y. P. et al., 2022), they are best understood as weighted risk factors that can be compounded in what is described as oligogenic ALS (Mejzini et al., 2019). One study has shown that the rate of an individual ALS patient harboring more than one ALS-related gene mutation was found to be low (3.8%), and would struggle by itself to fully explain rates of FALS (Cady et al., 2015). The environmental cause is thus likely a major contributing factor and will be the topic of focus in the following sections. A recent review that included over 258 studies has identified β-N-methylamino-L-alanine (BMAA), formaldehyde, mercury, manganese and zinc as key contributors to ALS risk in descending order of association (Newell et al., 2022). Other studies indicate herbicides and pesticides that include paraquat, permethrin and glyphosate as being associated risk factors (Malek et al., 2012; Andrew et al., 2021). BMAA is a cyanobacterial, algal and cycad plant neurotoxin that has been implicated in protein misfolding in neurodegenerative diseases including PD due to its mis-incorporation into translating proteins instead of serine that results in aggregation-prone proteins, as well as its glutamate excitotoxic effects. It also acts a strong chelator of copper, zinc and nickel (Nunn et al., 1989; Duncan et al., 1990; Lobner, 2009; Nunes-Costa et al., 2020). We speculate that when combined, this could enhance the copper binding and aggregation ability of BMAA mistranslated protein, although this should be experimentally proven. Furthermore, we also speculate that this chelation of copper by BMAA may be responsible for its enhanced ROS generation by acting as an aberrant BMAA-copper ROS inducing complex (Chiu et al., 2012). Interestingly, through comparing the works of Davis et al. (2020) and Hilton et al. (2024), it is evident that BMAA toxin-induced microglial activation in vervet monkey spinal cords lateral corticospinal tracts mirrors the activation areas evident in SALS, and these are also the areas in which there appears to be pathological copper accumulation, suggesting a link between copper disturbance and microglial activation in SALS (Figure 4). It would be interesting to determine if the same patterns of microglial activation and changes in copper levels are evident in samples from both these studies, and whether the copper is being increasingly localized to the microglia. Furthermore with regards to microglial activation, both human SALS patients and toxin-derived models of ALS in mice and these vervet monkeys display higher levels of spinal cord microglial activation (Kuo et al., 2019; Davis et al., 2020; Hilton et al., 2024). This activation could be reduced by delivery of copper using ionophores (Kuo et al., 2019), suggesting that sufficient metalation of microglial cuproenzymes could lead to a less toxic phenotype. Conversely, the excess of reactive copper within regions of ALS patient spinal cords and the potential loss of copper buffering ability of the astrocytes could lead to excess copper that is known to drive an inflammatory neurodegenerative microglial phenotype in an Alzheimer’s disease mouse model (Lim et al., 2020). Of course, both toxic excess and deficiency could be happening simultaneously in microglia, which will ultimately determine their phenotype. In addition to this, a recent study in mice has demonstrated that chronic administration of BMAA resulted in cytoplasmic TDP-43 accumulation with glial activation and ALS like symptoms (Anzilotti, 2023).

BMAA can enter the body via drinking water with algal contamination, a problem compounded by lack of testing and ineffectiveness of flocculation to remove this toxin (Cox et al., 2005; Holtcamp, 2012). Contaminated irrigation water has led to BMAA accumulation in crops without any obvious morphological alterations, making contamination hard to detect (Mohamed et al., 2024). It also has the potential to biomagnify further through the trophic levels and has been found in many fish species and in seafood sold in Swedish supermarkets (Jiang et al., 2014; Lopicic et al., 2022). Consumption of water and or fish from often algal bloom contaminated Lake Mascoma in New Hampshire has been suggested to play a large role in its almost 25 times higher annual incidence of ALS compared to the rest of the USA (Caller et al., 2009).

In addition to BMAA, formaldehyde crosslinks proteins that can denature them and is an identified inducer of protein aggregates. For instance, formaldehyde exposure induces Tau phosphorylation and Aβ accumulation in primates and also acts as a strong copper reductant (Byerley and Teo, 1969; Su et al., 2016; Tayri-Wilk et al., 2020). Interestingly BMAA is also metabolized into formaldehyde via methylamine as an intermediary, and thus a direct mechanistic link exists between the two greatest ALS environmental risk factors (Yu and Zuo, 1993; Spencer et al., 2012; Newell et al., 2022).

As discussed earlier, paraquat interacts with copper in ROS generation and induces TDP-43 mislocalisation, glyphosate increases prion aggregation and propagation *in vivo* and strongly binds to copper and zinc, acting as a chelator (Duke et al., 2012; Lafon et al., 2018). Heavy metals can also greatly interfere with copper and other metal ion balances by stressing the metal ion buffering systems, such as GSH and metallothioneins, as well as other enzymes that disable them via binding to the thiol groups of these proteins and thereby affect the cysteine residues, disrupting metalloprotein function and redox balance of the cell in addition to DNA damage (Balali-Mood et al., 2021). Furthermore, chronic zinc toxicity generally manifests as copper deficieny as these two metals have an interdependent relationship, (Harris, 2001; Agnew and Slesinger, 2022) and manganese is known to affect mitochondria and induce protein misfolding (Harischandra et al., 2019). Exposure to heavy metals and toxins may in part explain why ALS is more prevalent among males than females, as occupations such as heavy industrial work, agriculture or military service are generally considered male dominated industries (McCombe and Henderson, 2010; Gunnarsson and Bodin, 2018; McKay et al., 2021).

**Figure 5:**
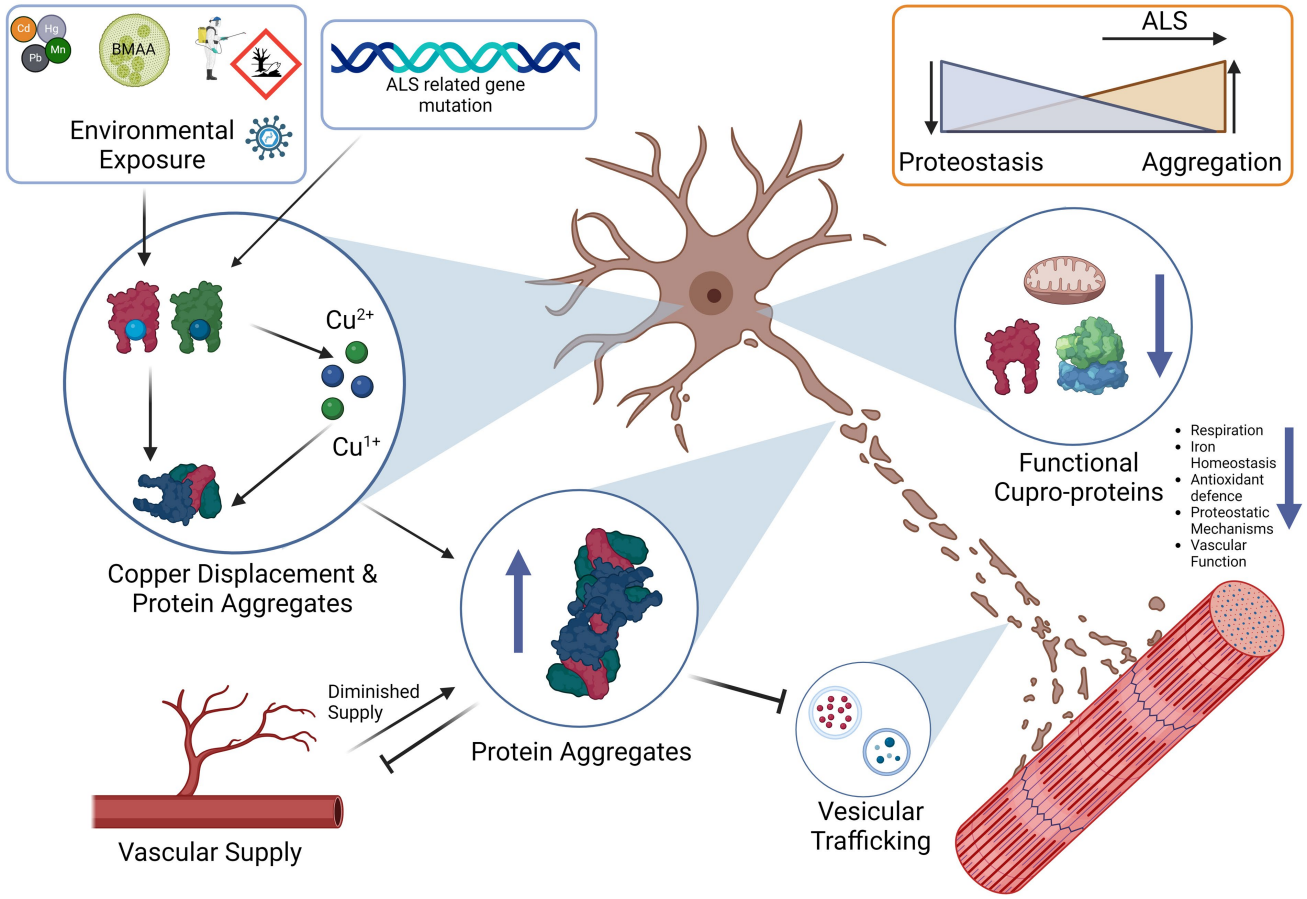
Proposed synthesis of pathological contributors to ALS and downstream consequences. Environmental exposure of toxins or viruses disrupts metal ion homeostasis and proteostasis resulting in displaced copper. Displaced copper could bind to proteins non-canonically and potentially enhance aggregation. ALS gene mutations can contribute by modifying proteins that affect protein stability and aggregation propensity. Protein aggregates disrupt vesicular trafficking of RNA, neurotransmitters and are a source of proteotoxic stress, these aggregates also reduce vascular supply. Overall cuproprotein function is decreased with effects such as defects in oxidative respiration, vascular signaling, antioxidant defense and other proteostatic mechanisms. The end result is the gradual decline and death of the motor neuron. Phenoconversion likely occurs when the rate of proteostasis is exceeded by the rate of protein aggregation and neuronal functions can no longer be optimally sustained.

Many of the environmental risk factors have the commonality of inducing protein aggregation, and also a close interaction with copper, mostly as a chelator, or else disrupting the metal ion buffering systems. It is plausible that these factors could be stripping copper from vital cuproenzymes, and at the same time contributing to the aggregative burden (Figure 5), as well as pressuring metal ion homeostasis. The findings of these studies are vital in demonstrating the relationship between these environmental and occupational toxins and are important in providing evidence for regulatory frameworks to mitigate exposure to such toxins.

Even with enhanced preventative measures it would still be extremely difficult to reduce all incidence of ALS, and so treatment is of great importance. One treatment modality with great potential is the use of copper ionophore therapy. As yet the most studied drug is Cu-ATSM, which is currently used in clinical trials (Kernel Networks Inc, 2019; Nikseresht et al., 2020). Emerging drugs such as the antisense oligonucelotide Tofersen shows promise in SOD1 patients, but its mechanism of action runs the risk of systemically reducing SOD1 by encouraging SOD1 mRNA degradation (Miller et al., 2022). However, animal studies have shown that CuATSM alone only increases lifespan by up to 25%, and that the treatment of SOD1 mice with ATSM without copper failed to yield any benefits (Williams et al., 2016; Vieira et al., 2017). This is again an argument supporting copper deficiency as being a major factor, and the importance of introducing exogenous copper together with ATSM, as ATSM alone seems to be unable to mobilize existing copper stores to the necessary locations.

The only example that demonstrates a 500% increase in the lifespan is the use of CuATSM with overexpression of CCS in SOD1G93A mice (Williams et al., 2016). This combination highlights the core argument that adequate CNS delivery of copper, paired with a sufficient degree of molecular chaperoning to ensure proper loading of copper, is highly effective in promoting the lifespan of treated transgenic mice. CNS-permeable synthetic copper delivery agents such as CuATSM, CuGTSM, Copper-histidinate (CuHis), and copper-glycyl histidly lysine (GHK-Cu; Pickart et al., 2017) could be combined with CCS as a polytherapy (Lem et al., 2007; Pickart et al., 2017; Baguña Torres et al., 2019). This could be particularly important in ALS, as CCS is a significant survival-associated gene in ALS, with patients expressing high levels of CCS experiencing improved survival (Swindell et al., 2019). The use of plant-extracted or recombinant CCS has been previously proposed in the treatment of AD and other SOD-related diseases (Kastenholz et al., 2011). Interestingly, CCS also binds to the β-site AβPP cleaving enzyme (BACE1) and other neuronal proteins, indicating its importance as a multi-target chaperone and therefore as a potential target for other neurodegenerative diseases (Gray et al., 2010; Kastenholz et al., 2011).

From the molecular understanding of how CCS functions in SOD1 stabilization, HIF-1 and also Aβ processing, efforts have been made to identify CCS mimetics that can promote the proper disulfide bond required for SOD1 maturation. This possibly even extends to HIF-1 and BACE1 as they share the same chaperone. One molecule investigated to achieve this is the CNS bioavailable selenium drug Ebselen that has been demonstrated to synergise with CuATSM to promote disulfide bond formation in SOD1 in multiple SOD1 mutant cell lines (McAlary et al., 2022). As an added bonus, Ebselen has been well studied for its anti-inflammatory and antioxidant properties and this may synergise with the immune system in ALS patients (Sarma and Mugesh, 2008). The effect of such a synergy has yet to be studied *in vivo*.

## Potential treatment modalities

In SALS patient spinal cords there is a dramatic shift in copper distribution, with heavy copper depletion in the spinal cord gray matter and accumulation in the dorsolateral white matter. This was followed by a decrease in CP activity as well as a shift in soluble copper to the insoluble fraction (Hilton et al., 2024) which is consistent with the suggestion posited earlier of copper based aggregation occurring. Furthermore, copper could also be possibly accumulated within microglia, based upon the coincidence seen in Figure 4 in an insoluble format, although it is unclear whether this is an intercellular or intracellular shift. Administration of CuATSM in a non-genetic model neurotoxin model of ALS has also demonstrated that CuATSM is also effective in preventing motor neuron degeneration (Kuo et al., 2019). We therefore suggest from the information presented in this review that a copper ionophore polytherapy paired with a CCS mimetic would be of great interest not only potential treatment of SOD1 FALS patients, but also for SALS. More effort should be made to determine not only the crude levels of copper within the spinal cord, but also to examine the sub-anatomical distribution. Such studies could include both SALS and FALS spinal cord samples.

Experimentation in BMAA-challenged vervet monkeys indicates L-serine as a protective agent against BMAA-induced neuronal damage (Davis et al., 2020). Due to BMAA being mistranslated in lieu of serine, L-Serine supplementation has received interest as a treatment for ALS, resulting in completed phase 1 and phase 2 clinical trials (Clinical Trial NCT03580616; Levine et al., 2017). It would be of interest to test if both Cu-ATSM (possibly with Ebselen) and L-Serine could be combined in treating ALS.

To conclude, we implicate copper dyshomeostasis as a crucial element in ALS and discuss the various affected pathways. Primarily the weight of the evidence suggests that it is a functional deficiency of copper and the loss of critical cuproprotein function that is at the heart of ALS, although there is also evidence to suggest that toxicity may also play a role although the evidence here is less direct. However the end result appears to be a deficiency and mispartitioning of copper as seen in SALS spinal cords which suggests that this aspect of ALS should receive greater research attention. Furthermore, we discuss evidence for the environmental causes of ALS that may be key to understanding why this copper depletion occurs. Thus we recommend that increased monitoring of known environmental contributors to ALS risk such as occupational and dietary exposure to BMAA, formaldehyde, heavy metals and agrochemicals would be beneficial, as well as preventative measures to limit exposure to populations at risk. In addition, strategies to treat the progression of ALS such as L-serine- or Cu-ATSM-centered therapies could be further studied and even combined.

## Author contributions

J-HM: Conceptualization, Investigation, Writing – original draft, Writing – review & editing, Visualization. HS: Supervision, Writing – review & editing. RH: Funding acquisition, Project administration, Resources, Supervision, Writing – review & editing.
